# Experimental infections with Zika virus strains reveal high vector competence of *Aedes albopictus* and *Aedes aegypti* populations from Gabon (Central Africa) for the African virus lineage

**DOI:** 10.1080/22221751.2021.1939167

**Published:** 2021-06-18

**Authors:** Davy Jiolle, Isabelle Moltini-Conclois, Judicaël Obame-Nkoghe, Patrick Yangari, Angélique Porciani, Bethsabée Scheid, Pierre Kengne, Diego Ayala, Anna-Bella Failloux, Christophe Paupy

**Affiliations:** a MIVEGEC Laboratory, Montpellier University, IRD, CNRS, Montpellier, France; b Ecologie des Systèmes Vectoriels, Centre Interdisciplinaire de Recherches Médicales de Franceville, Franceville, Gabon; c Laboratoire de Biologie Moléculaire et Cellulaire, Département de Biologie, Université des Sciences et Techniques de Masuku, Franceville, Gabon; d Arboviruses and Insect Vectors Unit, Institut Pasteur, Paris, France

**Keywords:** Zika virus, African and Asian lineages, *Aedes aegypti*, *Aedes albopictus*, Gabon

## Abstract

The two main Zika virus (ZIKV) vectors, *Aedes albopictus* and *Aedes aegypti* (invasive and native species, respectively), are present in Gabon (Central Africa). The aim of this study was to determine the entomological ZIKV risk associated with these mosquito species in Gabon by evaluating their vector competence for an African (i.e. representative of the endemic strains circulating in sub-Saharan Africa) and two Asian (i.e. representatives of exogenous epidemic strains that could be introduced) ZIKV strains. The transmission efficiency of one *Ae. aegypti* and two *Ae. albopictus* field-collected populations from Libreville and Franceville was assayed at day 7, 14 and 21 after experimental oral infection. The two mosquito species could transmit all three ZIKV strains already at day 7 post-infection, but transmission efficiency was higher for the African strain than the non-African strains (>60% versus <14%; incubation period of 14–21 days). The two mosquito species exhibited comparable vector competence for ZIKV, although the amount of viral particles (African strain) in saliva was significantly higher in *Ae. albopictus* than *Ae. aegypti* at day 14 post-infection. These findings suggest that overall, ZIKV risk in Gabon is mainly related to virus strains that circulate endemically across sub-Saharan Africa, although the transmission of non-African strains remain possible in case of introduction. Due to its high infestation indexes and ecological/geographical ranges, this risk appears mainly associated with *Ae. albopictus*. Vector surveillance and control methods against this invasive mosquito must be strengthened in the region to limit the risk of future outbreaks.

## Introduction

The mosquito-borne Zika virus (ZIKV, *Flavivirus* genus) was first isolated in 1947 from a sentinel Rhesus monkey stationed in the Zika forest in Uganda (East Africa) and one year later from an *Aedes africanus* mosquito pool [1]. In this part of the world, ZIKV primarily circulates in enzootic cycles that involve wild vertebrates and sylvatic mosquitoes [2] throughout sub-Saharan Africa [3].

Phylogenetic studies suggested that independent secondary introductions have occurred from its geographic origin into West Africa and Asia during the first half of the twentieth century, leading to the subsequent differentiation of African and Asian lineages [4,5]. In 2007, ZIKV spread from Asia to the Western Pacific Islands of Yap, Federated States of Micronesia, where it caused the first notable human epidemic [6], and later to several South Pacific islands, including French Polynesia in 2013 [7]. The virus reached Brazil between 2013 and 2015 and then spread across the Americas and Caribbean [8]. The recent spread of the Asian ZIKV has caused a major epidemic with millions of infections (more than 130 million in the Americas) [9], and neurological complications in adults [10] and neonates (particularly microcephaly, due to congenital infection) [11].

Despite multiple serological evidences of ZIKV infection in humans throughout tropical Africa (reviewed in [3]), the viral circulation remained globally silent on the continent until 2015 when sporadic human ZIKV infections were reported [12]. However, recent retrospective serological studies suggested that unidentified outbreaks occurred in West Africa, for instance in semi-arid regions of Mali in the late 1990s [13] and in Senegal and Nigeria where ZIKV has been silently circulating between 1996 and 2015 [14]. The single major noticeable ZIKV African outbreak was recorded in 2015–2016 in the Cape Verde archipelago (7580 cases with the first description of ZIKV-associated microcephaly in Africa) [15], following the secondary introduction of the Asian genotype from Brazil [16]. ZIKV introduction from the Americas, followed by autochthonous transmission of the Asian ZIKV lineage, was also recorded in Angola in 2015–2016 where cases of ZIKV-associated microcephaly have been reported [17]. ZIKV-associated microcephaly has also been suspected in Guinea-Bissau, but the virus origin (African or Asian lineage) could not be determined [18]. Indeed, despite an increasing number of studies suggesting intrinsic differences in the pathogenicity/virulence between African and Asian ZIKV lineages, it is still complex to predict whether an African epidemic due to an African ZIKV strain might result in similar or more severe neurological symptoms compared with what observed in South America [19–22]. As both lineages might now circulate in Africa, it is crucial to determine whether they can cause major epidemics in urban settings by assessing their transmissibility by urban local *Aedes* mosquito vectors. Indeed, African populations of *Aedes aegypti* display a lower ZIKV transmission potential than out-of-Africa populations, and this could have hindered ZIKV emergence on the African continent [23].

In Central Africa, ZIKV circulation was suggested by several serological studies carried out in Cameroon, Central African Republic and Gabon between the 1960s and 1980s (reviewed in [3]), and the virus is considered to be endemic in all sub-Saharan Africa. This was recently confirmed by serological findings from Cameroon [24]. However, this study suggested low circulation levels in urban settings and the probable existence of a (peri-)sylvatic cycle of ZIKV transmission rather than an urban “dengue-like” transmission. Together with other data from Democratic Republic of Congo [25], these findings suggest that overall, the immunity against ZIKV is low in Central African human populations, particularly in urban settings, thus highlighting the potential risk of epidemic spread. An active circulation of the African ZIKV genotype was detected in humans in Gabon in 2007, but remained restricted to Libreville and Cocobeach, two coastal cities [26]. Moreover, the virus was found in *Aedes albopictus* pools collected in Libreville, suggesting that this invasive mosquito species may be the primary epidemic vector, especially because ZIKV was not recovered in pools of the African native mosquito *Ae. aegypti* [26].

*Aedes aegypti*, which is considered the main ZIKV vector worldwide [3], is a native species in cities of Central Africa, but has been quickly declining following *Ae*. *albopictus* introduction in the 2000s [27]. Except in the drier northern parts of the region and in highly structured urban habitats, *Ae. albopictus* has widely outnumbered *Ae. aegypti* to became the dominant species in cities [27–29] and in remote forested locations [30]. Although *Ae. albopictus* is considered a less efficient laboratory ZIKV vector compared with *Ae. aegypti* in Asia [31] and Americas [32], it is mandatory to evaluate the vector competence (VC) for ZIKV of both species in Central Africa to better estimate their epidemical threat. It was recently reported that *Ae*. *aegypti* and *Ae*. *albopictus* (to a lower extent) populations from Cameroon and Republic of Congo can transmit African ZIKV strains [33]. Here, to better take into account the diversity of ZIKV strains that could be imported into urban areas in Central Africa, we assessed the VC of *Ae*. *aegypti* and *Ae*. *albopictus* sampled in Gabon for three ZIKV strains (African and Asian lineages).

## Material and methods

### Ethical statement

Mosquitoes were collected in Gabon under the research authorization AR0013/17/MESRS/CENAREST/CG/CST/CSAR delivered by CENASREST. IRD is accredited by the French Ministry of Higher Education and Research and Innovation to perform experiments on live animals in compliance with the French and European regulations on the care and protection of laboratory animals (agreement number: E34172221).

### Mosquito populations

Wild eggs (i.e. F0 generation) of *Aedes* mosquitoes were collected using ovitraps deployed in five sites of Franceville and in three sites of Libreville, Gabon (see supplemental data details). Dried eggs were shipped to IRD, Montpellier for hatching and mosquito rearing in controlled insectary conditions (28°C, 80% relative humidity, 14:10 hour light-dark cycle). Emerged adults were then mixed to establish three composite populations: *Ae. albopictus* from Franceville (FCV), *Ae. aegypti* from Franceville and *Ae. albopictus* from Libreville (LBV). Adult females were fed with rabbit blood to obtain eggs (i.e. F1 generation) used for experimental infections of *Ae. albopictus* colonies (-FCV and -LBV). The *Ae. aegypti*-FCV population was amplified over one additional generation and experiments were done on F2. A previous genetic analysis established that *Ae. aegypti* in Franceville fit with the “Aaf” form that encompasses most of the sub-Sahara African populations and is genetically distinct from the cosmopolitan “Aaa” form [34].

### ZIKV strains

The three mosquito populations were orally infected with three ZIKV strains provided by EVAg (https://www.european-virus-archive.com/) (see Tables S1 and S2 that summarizes the viral non-structural protein 1, NS1, with variations identified in the three strains). The Senegal strain (African, DAK84: *Ae. taylori*-tc/SEN/1984/41662-DAK) was isolated from an *Aedes taylori* mosquito pool collected in Senegal (GenBank accession number: KU955592) in December 1984. The Martinique strain (Asian, MARTI: MRS_OPY_Martinique_PaRi_2015) was isolated from a human serum in La Martinique (GenBank accession number: KU647676) in December 2015. The Malaysia strain (Asian, MAS66: ZIKV/*Aedes* aegypti/MYS/P6-740/1966) was isolated from an *Ae. aegypti* mosquito pool in Malaysia (GenBank accession number: KX694533.2) in January 1966.

### Experimental infections

All experimental infections were done in a BSL3 laboratory (IRD Vectopôle, Montpellier, France). Before infection, eggs were hatched synchronously by placing them at low pressure for 1 hour. Larvae were reared with a standard diet of yeast (Gayelord Hauser, France) in 24 × 34 × 9 cm plastic trays at a density of about 200 larvae per tray in 2 L of osmotic water. Adult mosquitoes (7-day-old females) were deprived of sucrose solution for 24 hours before experimental infection in which females were allowed to take an infectious blood meal (i.e. two volumes of washed rabbit erythrocytes and one volume of viral suspension at the final concentration of 1 × 10^6^ PFU/mL) through a section of desalted porcine intestine placed on a 37°C blood heater system (Hemotek) for 15 min. The viral concentration (1 × 10^6^ PFU/mL) is lower than what usually employed for dengue experimental infections (i.e. 1 × 10^7^ FFU/mL), but better fits with the ZIKV loads observed in human blood samples (i.e. about 100-fold lower than in viraemic blood samples from patients with dengue) [8,35,36]. Adenosine triphosphate was added to the blood meal as phagostimulant at the final concentration of 10 mM. Rabbit erythrocytes were obtained from arterial blood collected and washed the day before the experimental infection. Fully engorged females were sorted on ice and incubated in groups of about 30 females in controlled conditions (28 ± 1°C, 80% relative humidity, 14:10 hour light-dark cycle). At 7, 14 and 21 days post-infection (dpi), females were anesthetized on ice to remove wings and legs and to collect saliva by inserting the proboscis in a 20 µL tip containing 5 µL of FBS. After 30 min, saliva was transferred in a well of a 96-well plate with 45 µL DMEM (Dulbecco's Modified Eagle Medium). Then, mosquito heads and bodies were separated on ice and put individually in 2 mL tubes containing glass beads and 300 µL of DMEM/2% FBS (Fetal Bovine Serum). Tubes and plates containing samples were stored at −80°C until processing. For each viral strain, 30 females of each mosquito population were examined at 7, 14 and 21 dpi.

### Virus detection/quantification

A plaque assay technique with Vero cells was used to determine ZIKV presence in bodies and heads (see supplemental data for a detailed protocol). ZIKV presence in saliva was determined by inoculating 25 µL of saliva in 6-well plates, and by counting foci by the naked eye and converting them into PFU/saliva. These data were then used to estimate the following parameters to characterize the VC. Infection rate (IR) is the proportion of mosquitoes with infected body (abdomen and thorax) among the initial number of females tested (INFT). Dissemination efficiency (DE) and transmission efficiency (TE) are the proportion of mosquitoes with infected head and with infectious saliva among all INTF, respectively [37]. Finally, the saliva viral titre for each mosquito was estimated to determine the number of excreted viral particles.

### Statistical analysis

All statistical analyses were performed with the R software, version 4.0.0 [38]. The percentages of infected mosquitoes, of infected mosquitoes with disseminated infection, and of infected mosquitoes with a disseminated infection and positive saliva were assessed by logistic regression analysis using the bias-reduction method and the “brglm” package [39]. Tuckey pairwise comparisons were done with the “emmeans” package [40]. Quantitative variables were expressed by means and compared using the Kruskal-Wallis non-parametric test. Multiple pairwise comparisons were done with the Dunn test and p-values were adjusted with the Benjamini-Hochberg method. A *P value <* 0.05 was considered significant.

## Results

The African ZIKV strain is better transmitted by *Ae. aegypti* and *Ae. albopictus* from Gabon compared with the strains from Malaysia and Martinique

**Figure 1.**
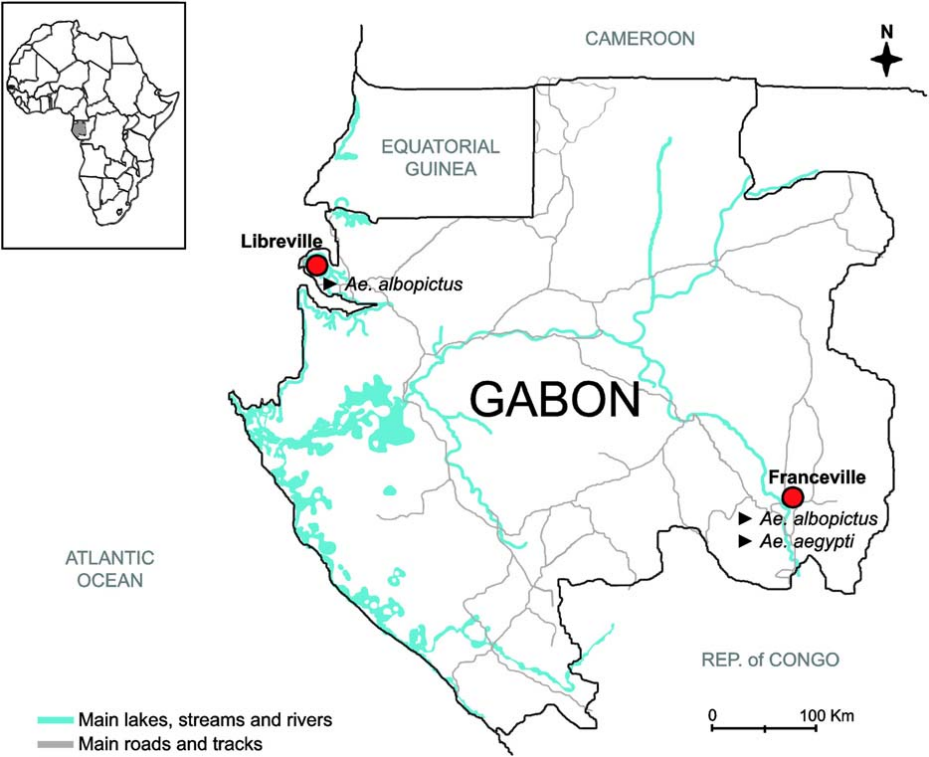
*Ae. albopictus* and *Ae. aegypti* sampling sites in Gabon, Central Africa.

**Figure 2.**
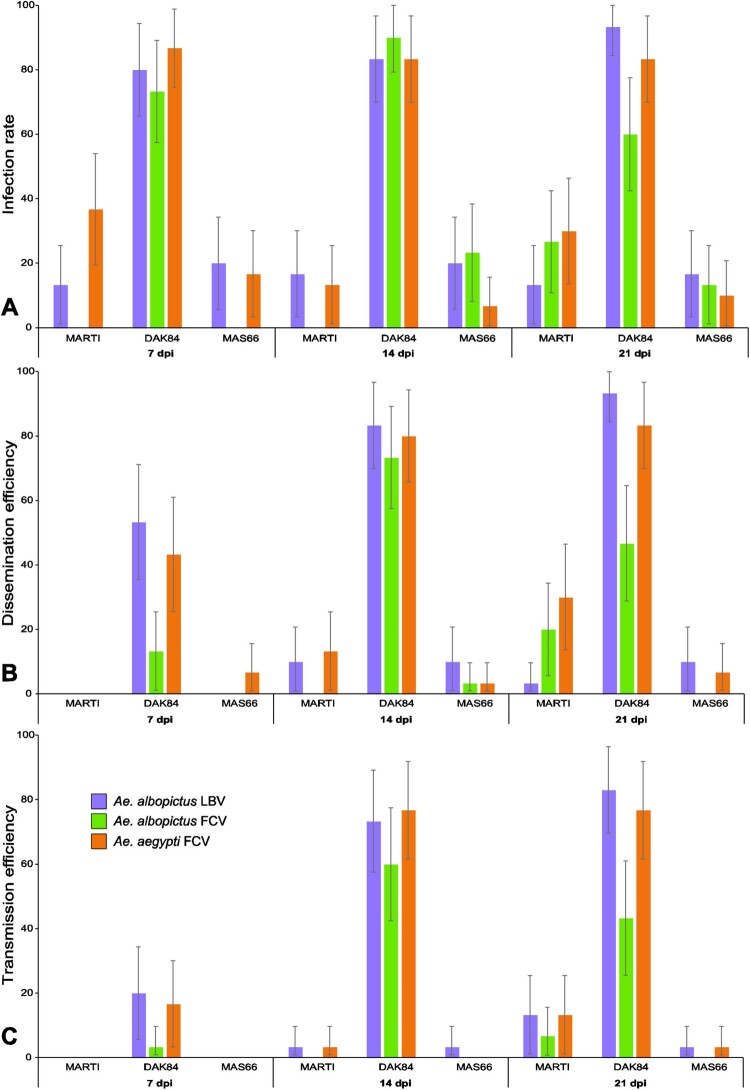
Infection rate (±95% CI) (A), dissemination efficiency (±95% CI) (B), and transmission efficiency (±95% CI) (C) at day 7, 14 and 21 post-infection (dpi) with three ZIKV strains (MARTI, DAK84 and MASS66) in *Ae. albopictus* and *Ae. aegypti* from Libreville (LBV) and Franceville (FCV). For each modality, a batch of 30 mosquitoes was analyzed (see Table S1 for the statistical tests).

The VC of one *Ae. aegypti* and two *Ae. albopictus* populations from Gabon (Figure 1) for ZIKV strains of the African (DAK84) and Asian (MAS66 and MARTI) genotypes was assessed by quantifying several parameters at 7, 14 and 21 dpi. The IR values of the DAK84 strain were significantly higher than those of the two Asian ZIKV strains in all mosquito populations and at all-time points (Figure 2, Tables 1 and S3). At 7, 14 and 21 dpi, the IR values for the DAK84 strain were [73.3–86.7], [83.3–90.0], and [60.0–93.3] for *Ae. albopictus*-LBV, *Ae. albopictus*-FCV, and *Ae. aegypti*-FCV, respectively (Table 1), [0.0–36.7], [0.0–16.7] and [13.3–30.0] for the MARTI, and [0.0–20.0], [6.7–23.3] and [10.0–16.7] for the MAS66 strain, without difference between the Asian genotypes.

**Table 1.** Infection rate, dissemination and transmission efficiency for *Aedes albopictus* (Franceville and Libreville) and *Aedes aegypti* (Franceville) populations from Gabon at day 7, 14 and 21 post-infection with three different ZIKV strains.

		Infection rates (%)			Dissemination efficiency (%)			Transmission efficiency (%)		
		Day post-infection			Day post-infection			Day post-infection		
Mosquito population	ZIKV strain	*7*	*14*	*21*	*7*	*14*	*21*	*7*	*14*	*21*
*Ae. albopictus-*LBV	MARTI	13.3 (30)	16.7 (30)	13.3 (30)	0 (30)	10 (30)	13.3 (30)	0 (30)	3.3 (30)	13.3 (30)
	MAS66	20 (30)	20 (30)	16.7 (30)	0 (30)	10 (30)	10 (30)	0 (30)	3.3 (30)	3.3 (30)
	DAK84	80 (30)	83.3 (30)	93.3 (30)	53.3 (30)	83.3 (30)	93.3 (30)	20 (30)	73.3 (30)	83 (30)
*Ae. albopictus-*FCV	MARTI	0 (30)	0 (30)	26.7 (30)	0 (30)	0 (30)	20 (30)	0 (30)	0 (30)	6.7 (30)
	MAS66	0 (30)	23.3 (30)	13.3 (30)	0 (30)	3.3 (30)	0 (30)	0 (30)	0 (30)	0 (30)
	DAK84	73.3 (30)	90 (30)	60 (30)	13.3 (30)	73.3 (30)	46.7 (30)	3,3 (30)	60 (30)	43.3 (30)
*Ae. aegypti-*FCV	MARTI	36.7 (30)	13.3 (30)	30 (30)	0 (30)	13.3 (30)	30 (30)	0 (30)	3.3 (30)	13.3 (30)
	MAS66	16.7 (30)	6.7 (30)	10 (30)	6.7 (30)	3.3 (30)	6.7 (30)	0 (30)	0 (30)	3.3 (30)
	DAK84	86.7 (30)	83.3 (30)	83.3 (30)	43.3 (30)	80 (30)	83.3 (30)	16.7 (30)	76.7 (30)	76.7 (30)

Note: Numbers in parentheses correspond to the number of analyzed mosquitoes.

With two exceptions (i.e. the DAK84-MARTI pair, for which no statistical difference was detected in *Ae. albopictus*-FCV at 7 and 21 dpi), the DE values also were significantly higher after infections with the DAK84 than with the MARTI and MAS66 strains (Figure 2, Table S3). The DE values were [13.3–53.3], [73.3–83.3] and [46.7–93.3] for DAK84 at 7, 14 and 21 dpi, [0.0–13.3] and [13.3–30.0] for MARTI at 14 and 21 dpi (no detectable viral dissemination at 7 dpi), and [0.0–6.7], [3.3–10.0] and [0.0–10.0] for MAS66 (Table 1). No statistical difference was detected between the DE values of the two Asian ZIKV genotypes at all-time points and with all mosquito populations.

Finally, the TE values for the DAK84 strain were [3.3–20.0], [60.0–76.7], and [43.3–83.3] at 7, 14, 21 dpi, respectively (Table 1) and were significantly higher than for MARTI and MAS66 strains (Table S3). No transmission was detected before 14 dpi for the Asian ZIKV strains whatever the mosquito population tested, with very low TE values (3.3% in *Ae. albopictus*-LBV infected with the MARTI and MAS66 strains, and in *Ae. aegypti*-FCV infected with the MARTI strain). At 21 dpi, the TE values for all mosquito populations remained very low after infection with the MARTI (6.7 to 13.3) and MAS66 (0 to 3.3) strains. No statistical difference was detected between the Asian ZIKV strains (Table S3).

**Figure 3.**
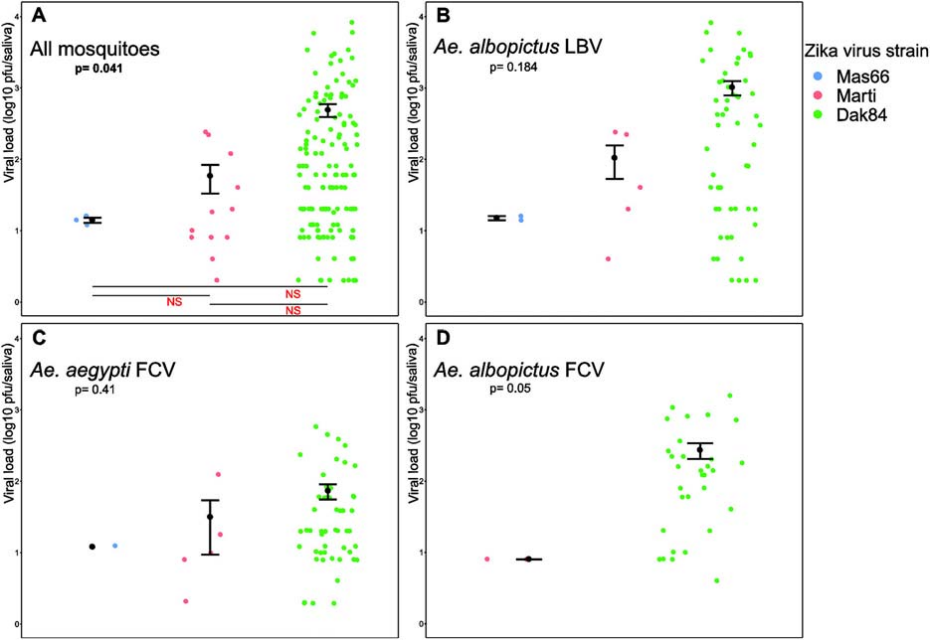
Viral loads in the saliva of mosquitoes infected with ZIKV strains from Malaysia (MAS66), Martinique (MARTI), and Senegal (DAK84), all days post-infection combined. Each coloured dot represents the titre of one saliva sample. Black dots and vertical bars represent the mean (±SEM). Viral loads are shown for all mosquito combined (A) and for the three populations (B), (C) and (D); p: probability associated with the global test (Kruskal-Wallis) to compare the mean viral loads obtained with the different ZIKV strains (in bold when <0.05). Horizontal bars and the associated annotation (“NS”, not significant) correspond to pairwise comparisons of the mean viral loads (Dunn test) when the global test was significant.

When all time points and mosquito populations were grouped, the mean ZIKV viral loads (expressed in log10 pfu/saliva extract) estimated in individual saliva samples were 1.15 ± 0.06 for the MAS66, 1.76 ± 1.40 for the MARTI, and 2.69 ± 2.00 for the DAK84 strain (Figure 3(A)). Although the global test (Kruskal-Wallis, *P* = 0.041) indicated a significant difference of mean viral loads in function of the ZIKV strain, pairwise comparisons of the mean viral loads (Dunn test) did not confirm this difference. Considering each mosquito population independently (Figure 3(B–D)), no statistical difference was detected among ZIKV strains.

These results clearly indicate that compared with the two Asian strains, the ZIKV strain of the African genotype displayed higher replication and transmission efficiency in *Ae. aegypti* and *Ae. albopictus,* two epidemic vector species in Gabon.

### *Ae. albopictus* and *Ae. aegypti* from Gabon exhibit comparable vector competence for ZIKV

After experimental infections with the MARTI and MAS66 ZIKV strains, all VC parameters (i.e. IR, DE and TE, Tables 1 and S3, Figure 2) and viral loads in saliva (Figure S1) were similar among mosquito populations and time points. After experimental infection with the DAK84 strain, DE at 7 dpi, and IR, DE and TE at 21 dpi, were significantly lower in *Ae. albopictus*-FCV than in *Ae. albopictus*-LBV and *Ae. aegypti*-FCV. Although all these parameters were similar among mosquito strains at 14 dpi, these results suggest a lower ability of *Ae. albopictus*-FCV to transmit the African ZIKV strain at 21 dpi. Quantification of the viral loads in saliva after DAK84 infection (Figure S1) showed that viral load was lower in *Ae. aegypti*-FCV than in *Ae. albopictus*-FCV and -LBV at 14 dpi, and *Ae. albopictus*-LBV at 21 dpi. Despite some minor differences at 14 and 21 dpi, these results suggest that in our experimental conditions, the two mosquito species from Gabon show similar ability to transmit ZIKV: very efficiently for the African ZIKV and moderately efficiently for non-African ZIKV lineages.

## Discussion

Using a standardized protocol for oral experimental infections, we evaluated the infectiousness of three ZIKV strains (i.e. the African DAK84, and the Asian MARTI and MAS66) in the two main urban *Aedes* vectors species in Gabon. Our data indicate that *Ae. aegypti* and *Ae. albopictus* very efficiently transmitted the African ZIKV strain, already at 7 dpi, confirming previous observations made in mosquito populations from Cameroon and Republic of Congo artificially infected with DAK84 [33]. Moreover, compared with the two non-African ZIKV strains tested, DAK84 displayed a greater ability to infect and disseminate and to be transmitted by both vector species. This is in agreement with previous studies on *Ae. aegypti* [37,41,42] and *Ae. albopictus* [43–46] populations from the Americas, Europe and Pacific Ocean Islands [37,44–46]. Although DAK84 strong infectiousness and transmissibility are a general trend that does not depend on the vector species (*Ae. aegypti* vs. *Ae. albopictus*) or geographical origin (LBV vs. FCV), our findings highlight the strong adaptation of this African ZIKV strain to both *Ae. aegypti* and *Ae. albopictus* populations in Gabon. A recent study on different African *Ae. aegypti* populations (including one from Gabon) and several ZIKV strains also found higher infectiousness of an African ZIKV strain (isolated in Senegal in 2011) compared with non-African strains [23]. Moreover, it has been shown that some amino acid substitutions in NS1 significantly affect ZIKV infectiousness for *Ae. aegypti* and *Ae. albopictus* in a species-specific manner [47,48]. The comparison of the polyprotein amino acid sequences of the DAK84, MAS66 and MARTI strains indicated that three residues in NS1 (positions 988, 1007 and 1030; Table S2; Figure S2) differ in the African ZIKV strain compared with the two Asian strains. More investigation is needed to determine whether these differences might explain the stronger infectiousness of DAK84 for both *Aedes* mosquito species. Additional studies to cover the extent of the African ZIKV lineage diversity are needed to determine whether this observation can be generalized to other ZIKV African strains, and also to better assess the risk of emergence for the strains that are currently circulating silently in enzootic cycles or in African human populations. It was recently suggested that epidemics caused by African ZIKV strains might be less easily detected than those caused by Asian strains due to their propensity to cause foetal loss rather than birth defects [49].

We found that *Ae. aegypti* and *Ae. albopictus* populations from Gabon are competent for both ZIKV strains of the Asian lineage (MARTI and MAS66), but with lower VC parameters and TE values of 3.3% at 14 dpi and 13.3% at 21 dpi. This could be interpreted as a sign of poor adaptation of recent ZIKV epidemic strains from America and Asia to urban Gabonese *Aedes* populations, and consequently as a limited epidemic risk in Gabon. However, this might not fully correspond to the field conditions. Indeed, the viral load used to infect mosquitoes (1 × 10^6^ PFU/mL) was within the lower limit of those used in similar studies. We chose this load because it better fits with the viraemia observed in humans [50], but several studies demonstrated a positive relationship between viral dose and mosquito susceptibility to infection, with significant differences even for limited viral load increases [23,50,51]. Furthermore, it is worth emphasizing that membrane feeding systems significantly underestimate VC compared with *in vivo* models, as recently suggested by data obtained in ZIKV-infected mice [23,51]. Second, some mosquito populations, with similar or lower laboratory TE levels, allowed the rapid ZIKV spread, notably across American territories from 2015 to 2017. For example, *Ae. aegypti* populations in Guadeloupe and Cuba readily spread the “American strain” (represented by the MARTI in the present study) locally, although they display low laboratory TE for this viral strain [37,42]. Indeed other key parameters that modulate the vector capacity (e.g. vector density and longevity, aggressiveness for humans) may counterbalance a low VC to result in a very efficient epidemic transmission in field conditions [52].

As observed in neighbouring countries, *Ae. aegypti* has become uncommon in most urban environments of Gabon where it has been replaced by *Ae. albopictus* [53]. Although the native species remains dominant in the densely crowed central urban habitats of Libreville, its current geographic range and densities across the country have drastically reduced its possibility of contact with humans and therefore the risk for arbovirus transmission. Consequently, the potential role of *Ae. aegypti* as epidemic vector of ZIKV in Gabon appears limited for the African ZIKV lineage, and very limited for the Asian/American lineages because the vector density is too low to offset the limited VC observed with MARTI, MASS66 and other ZIKV strains [23]. Conversely, *Ae. albopictus* role as epidemic vector of ZIKV in Gabon might be more important because it is present in all human settings countrywide (and in forested regions of the bordering countries) with high human biting rates [30,53].

Overall, our results indicated similar TE levels for *Ae. aegypti* and *Ae. albopictus* populations from Gabon whatever the ZIKV lineage. In Gabon, like in most Central African countries, *Ae. aegypti* belongs to the native “Aaf” form that transmits ZIKV less efficient than the cosmopolitan invasive “Aaa” form from which is genetically distinct [23,34]. Therefore, the comparison between *Ae. aegypti* and *Ae. albopictus* made in the present study cannot be transposed to countries where the two species co-occurs in sympatry, because only the “Aaa” form exists outside Africa. Our results are globally consistent with recent VC data obtained for mosquito populations from Cameroon and Republic of Congo with the DAK84 ZIKV strain using a higher infectious titre, although the authors found a slight advantage for *Ae. aegypti* populations, while noting a significant geographic variability for this species and not for *Ae. albopictus* [33]. In our study, the amount of DAK84 ZIKV particles excreted in saliva was significantly higher for *Ae. albopictus* than for *Ae. aegypti* at 14 dpi. Although this parameter might vary in function of the geography, this difference between mosquito species could be related to the amount of viral particles accumulated in salivary glands, to the quantity of excreted saliva, or at equal saliva excretion volumes, to differences in the number of viral particles excreted in the saliva due to a stronger salivary gland escape barrier, as recently highlighted in *Ae. aegypti* (“Aaa” form from Mexico) infected with ZIKV [54]. How these differences in the quantity of viral particles delivered in saliva can be interpreted in terms of TE is not clear, but it was recently proposed the inoculum dose could influence ZIKV dynamics in non-human primates [55].

We observed significant VC differences (DE at 7 and 21 dpi, and TE at 21 dpi) between *Ae. albopictus-*LBV and -FCV after experimental infection with the African ZIKV strain, in agreement with the variations observed at the population level at different local geographical scales [33,44,45]. Nevertheless, our results clearly indicated that *Ae. albopictus* from Gabon can efficiently transmit an African ZIKV strain, corroborating previous field observations and viral detections in mosquito pools collected in 2007 during a ZIKV epidemic [26]. The TE levels at 14 or 21 dpi, the number of viral particles delivered in saliva, and the very high infestation indexes and anthropophilia [53] confirm that *Ae. albopictus* might establish a sustainable epidemic transmission of African ZIKV strains in Gabon and in Central Africa. Conversely, it is unclear how *Ae. albopictus* might support the epidemic activity by Asian ZIKV strains, although it could mediate their sporadic autochthonous transmission with similar VC in the South of France [56].

In conclusion, *Ae. aegypti* and *Ae. albopictus* from Gabon can transmit African and Asian ZIKV strains. Nevertheless, the higher VC and shorter extrinsic incubation period in both mosquito species for the strain belonging to the African lineage suggest a particularly high transmission risk and epidemic potential in Central Africa related to autochthonous rather than to non-native ZIKV. Due to its high infestation indexes and its current ecological and geographical ranges in the region, this risk appears mainly associated with *Ae. albopictus*. Therefore, vector surveillance and control methods against this invasive mosquito must be amplified in the region to mitigate the risk of future outbreaks.

## Supplementary Material

Figure_S2_editable.docx

Figure_S1_editable.eps

Supplementary_information_Jiolle_et_al._r_vis_e_clean_file.docx
