# Supplementary material for: Experimental infections with Zika virus strains reveal high vector competence of Aedes albopictus and Aedes aegypti populations from Gabon (Central Africa) for the African virus lineage
Source: Emerg Microbes Infect. 2021 Jun 18;10(1):1244–53. doi: 10.1080/22221751.2021.1939167 (PMC8216262; doi:10.1080/22221751.2021.1939167)
Supplement: Figure_S2_editable.docx [file TEMI_A_1939167_SM6363.docx]

**(1): MARTI**

| Alignment: /Users/cdauga/Desktop/Zika/proteins_zika.txt.aln  Seaview [blocks=10 fontsize=10 A4-landscape] on Mon May 10 08:25:42 2021 | **(2): DAK84** |
| --- | --- |

**(3): MAS66**

1

# **(1)**KU647676_AMC33116.1 MKNPKKKSGG FRIVNMLKRG VARVSPFGGL KRLPAGLLLG HGPIRMVLAI LAFLRFTAIK PSLGLINRWG SVGKKEAMEI IKKFKKDLAA MLRIINARKE


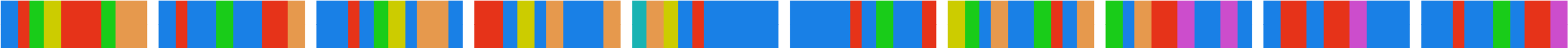
**(2)**KU955592_AMR39833.1 MKNPKKKSGG FRIVNMLKRG VARVNPLGGL KRLPAGLLLG HGPIRMVLAI LAFLRFTAIK PSLGLINRWG SVGKKEAMEI IKKFKKDLAA MLRIINARKE **(3)**KX694533_AOC50653.1 MKNPKKKSGG FRIVNMLKRG VARVSPFGGL KRLPAGLLLG HGPIRMVLAI LAFLRFTAIK PSLGLINRWG SVGKKEAMEI IKKFKKDLAA MLRIINARKE

# 101 KU647676_AMC33116.1 KKRRGAETSV GIVGLLLTTA MAAEVTRRGS AYYMYLDRND AGEAISFPTT LGMNKCYIQI MDLGHMCDAT MSYECPMLDE GVEPDDVDCW CNTTSTWVVY


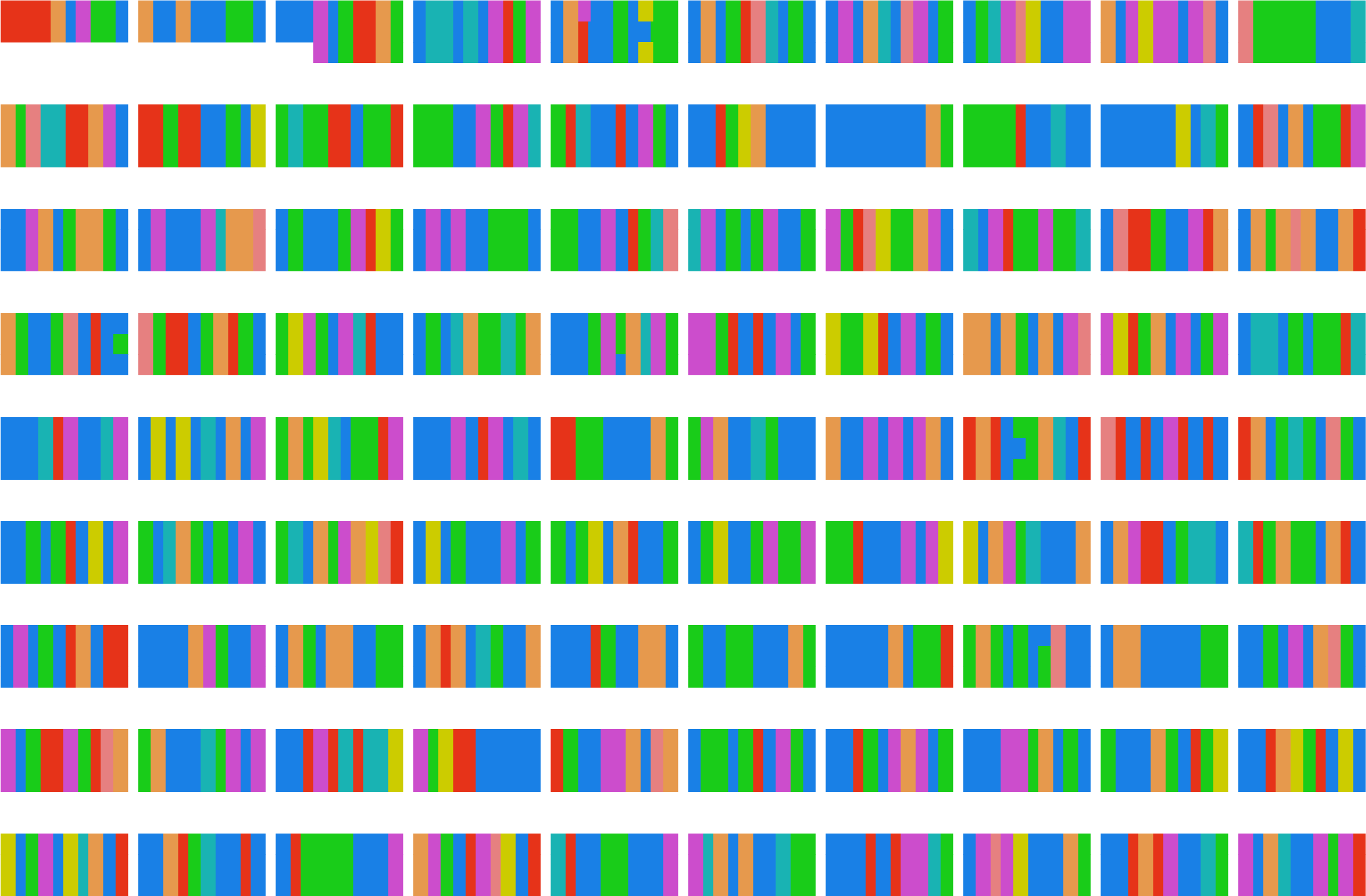
KU955592_AMR39833.1 RKRRGADTSI GIVGLLLTTA MAAEITRRGS AYYMYLDRSD AGKAISFATT LGVNKCHVQI MDLGHMCDAT MSYECPMLDE GVEPDDVDCW CNTTSTWVVY KX694533_AOC50653.1 KKRRGADTSV GIVGLLLTTA MAVEVTRRGS AYYMYLDRSD AGKAISFPTT LGVNKCYIQI MDLGHMCDAT MSYECPMLDE GVEPDDVDCW CNTTSTWVVY

# 201 KU647676_AMC33116.1 GTCHHKKGEA RRSRRAVTLP SHSTRKLQTR SQTWLESREY TKHLIRVENW IFRNPGFALA AAAIAWLLGS STSQKVIYLV MILLIAPAYS IRCIGVSNRD

KU955592_AMR39833.1 GTCHHKKGEA RRSRRAVTLP SHSTRKLQTR SQTWLESREY TKHLIKVENW IFRNPGFALV AVAIAWLLGS STSQKVIYLV MILLIAPAYS IRCIGVSNRD KX694533_AOC50653.1 GTCHHKKGEA RRSRRAVTLP SHSTRKLQTR SQTWLESREY TKHLIRVENW IFRNPGFALA AAAIAWLLGS STSQKVIYLV MILLIAPAYS IRCIGVSNRD

# 301 KU647676_AMC33116.1 FVEGMSGGTW VDVVLEHGGC VTVMAQDKPT VDIELVTTTV SNMAEVRSYC YEASISDMAS DSRCPTQGEA YLDKQSDTQY VCKRTLVDRG WGNGCGLFGK

KU955592_AMR39833.1 FVEGMSGGTW VDVVLEHGGC VTVMAQDKPT VDIELVTTTV SNMAEVRSYC YEASISDMAS DSRCPTQGEA YLDKQSDTQY VCKRTLVDRG WGNGCGLFGK KX694533_AOC50653.1 FVEGMSGGTW VDVVLEHGGC VTVMAQDKPT VDIELVTTTV SNMAEVRSYC YEASISDMAS DSRCPTQGEA YLDKQSDTQY VCKRTLVDRG WGNGCGLFGK

# 401 KU647676_AMC33116.1 GSLVTCAKFA CSKKMTGKSI QPENLEYRIM LSVHGSQHSG MIVNDTGHET DENRAKVEIT PNSPRAEATL GGFGSLGLDC EPRTGLDFSD LYYLTMNNKH

KU955592_AMR39833.1 GSLVTCAKFT CSKKMTGKSI QPENLEYRIM LSVHGSQHSG MIVNDTGHET DENRAKVEVT PNSPRAEATL GGFGSLGLDC EPRTGLDFSD LYYLTMNNKH KX694533_AOC50653.1 GSLVTCAKFA CSKKMTGKSI QPENLEYRIM LSVHGSQHSG MIVNDIGHET DENRAKVEIT PNSPRAEATL GGFGSLGLDC EPRTGLDFSD LYYLTMNNKH

# 501 KU647676_AMC33116.1 WLVHKEWFHD IPLPWHAGAD TGTPHWNNKE ALVEFKDAHA KRQTVVVLGS QEGAVHTALA GALEAEMDGA KGRLSSGHLK CRLKMDKLRL KGVSYSLCTA

KU955592_AMR39833.1 WLVHKEWFHD IPLPWHAGAD TGTPHWNNKE ALVEFKDAHA KRQTVVVLGS QEGAVHTALA GALEAEMDGA KGRLFSGHLK CRLKMDKLRL KGVSYSLCTA KX694533_AOC50653.1 WLVHKEWFHD IPLPWHAGAD TGTPHWNNKE ALVEFKDAHA KRQTVVVLGS QEGAVHTALA GALEAEMDGA KGRLSSGHLK CRLKMDKLRL KGVSYSLCTA

# 601 KU647676_AMC33116.1 AFTFTKIPAE TLHGTVTVEV QYAGTDGPCK VPAQMAVDMQ TLTPVGRLIT ANPVITESTE NSKMMLELDP PFGDSYIVIG VGEKKITHHW HRSGSTIGKA

KU955592_AMR39833.1 AFTFTKVPAE TLHGTVTVEV QYAGTDGPCK VPAQMAVDMQ TLTPVGRLIT ANPVITESTE NSKMMLELDP PFGDSYIVIG VGDKKITHHW HRSGSTIGKA KX694533_AOC50653.1 AFTFTKIPAE TLHGTVTVEV QYAGTDGPCK VPAQMAVDMQ TLTPVGRLIT ANPVITESTE NSKMMLELDP PFGDSYIVIG VGDKKITHHW HRSGSTIGKA

# 701 KU647676_AMC33116.1 FEATVRGAKR MAVLGDTAWD FGSVGGALNS LGKGIHQIFG AAFKSLFGGM SWFSQILIGT LLMWLGLNTK NGSISLMCLA LGGVLIFLST AVSADVGCSV

KU955592_AMR39833.1 FEATVRGAKR MAVLGDTAWD FGSVGGVFNS LGKGIHQIFG AAFKSLFGGM SWFSQILIGT LLVWLGLNTK NGSISLTCLA LGGVMIFLST AVSADVGCSV KX694533_AOC50653.1 FEATVRGAKR MAVLGDTAWD FGSVGGALNS LGKGIHQIFG AAFKSLFGGM SWFSQILIGT LLVWLGLNTK NGSISLTCLA LGGVLIFLST AVSADVGCSV

# 801 KU647676_AMC33116.1 DFSKKETRCG TGVFVYNDVE AWRDRYKYHP DSPRRLAAAV KQAWEDGICG ISSVSRMENI MWRSVEGELN AILEENGVQL TVVVGSVKNP MWRGPQRLPV

KU955592_AMR39833.1 DFSKKETRCG TGVFVYNDVE AWRDRYKYHP DSPRRLAAAV KQAWEEGICG ISSVSRMENI MWKSVEGELN AILEENGVQL TVVVGSVKNP MWRGPQRLPV KX694533_AOC50653.1 DFSKKETRCG TGVFVYNDVE AWRDRYKYHP DSPRRLAAAV KQAWEDGICG ISSVSRMENI MWRSVEGELN AILEENGVQL TVVVGSVKNP MWRGPQRLPV

# 901 *** *** KU647676_AMC33116.1 PVNELPHGWK AWGKSYFVRA AKTNNSFVVD GDTLKECPLK HRAWNSFLVE DHGFGVFHTS VWLKVREDYS LECDPAVIGT AVKGKEAVHS DLGYWIESEK KU955592_AMR39833.1 PVNELPHGWK AWGKSYFVRA AKTNNSFVVD GDTLKECPLK HRAWNSFLVE DHGFGIFHTS VWLKVREDYS LECDPAVIGT AVKGKEAAHS DLGYWIESEK KX694533_AOC50653.1 PVNELPHGWK AWGKSYFVRA AKTNNSFVVD GDTLKECPLK HRAWNSFLVE DHGFGVFHTS VWLKVREDYS LECDPAVIGT AAKGKEAVHS DLGYWIESEK 1001 *** *** KU647676_AMC33116.1 NDTWRLKRAH LIEMKTCEWP KSHTLWTDGI EESDLIIPKS LAGPLSHHNT REGYRTQMKG PWHSEELEIR FEECPGTKVH VEETCGTRGP SLRSTTASGR

KU955592_AMR39833.1 NDTWRLRRAH LIEMKTCEWP KSHTLWTDGV EESDLIIPKS LAGPLSHHNT REGYRTQVKG PWHSEELEIR FEECPGTKVH VEETCGTRGP SLRSTTASGR KX694533_AOC50653.1 NDTWRLKRAH LIEMKTCEWP KSHTLWTDGI EESDLIIPKS LAGPLSHHNT REGYRTQVKG PWHSEELEIR FEECPGTKVH VEETCGTRGP SLRSTTASGR

# 1101 ***** KU647676_AMC33116.1 VIEEWCCREC TMPPLSFWAK DGCWYGMEIR PRKEPESNLV RSMVTAGSTD HMDHFSLGVL VILLMVQEGL KKRMTTKIII STSMAVLVAM ILGGFSMSDL

KU955592_AMR39833.1 VIEEWCCREC TMPPLSFRAK DGCWYGMEIR PRKEPESNLV RSMVTAGSTD HMDHFSLGVL VILLMVQEGL KKRMTTKIIM STSMAVLVAM VLGGFSMSDL KX694533_AOC50653.1 VIEEWCCREC TMPPLSFRAK DGCWYGMEIR PRKEPESNLV RSMVTAGSTD HMDHFSLGVL VILLMVQEGL KKRMTTKIII STSMAVLVAM ILGGFSMSDL

# 1201 KU647676_AMC33116.1 AKLAILMGAT FAEMNTGGDV AHLALTAAFK VRPALLVSFI FRANWTPRES MLLALASCLL QTAISALEGD LMVLINGFAL AWLAIRAMVV PRTDNITLAI


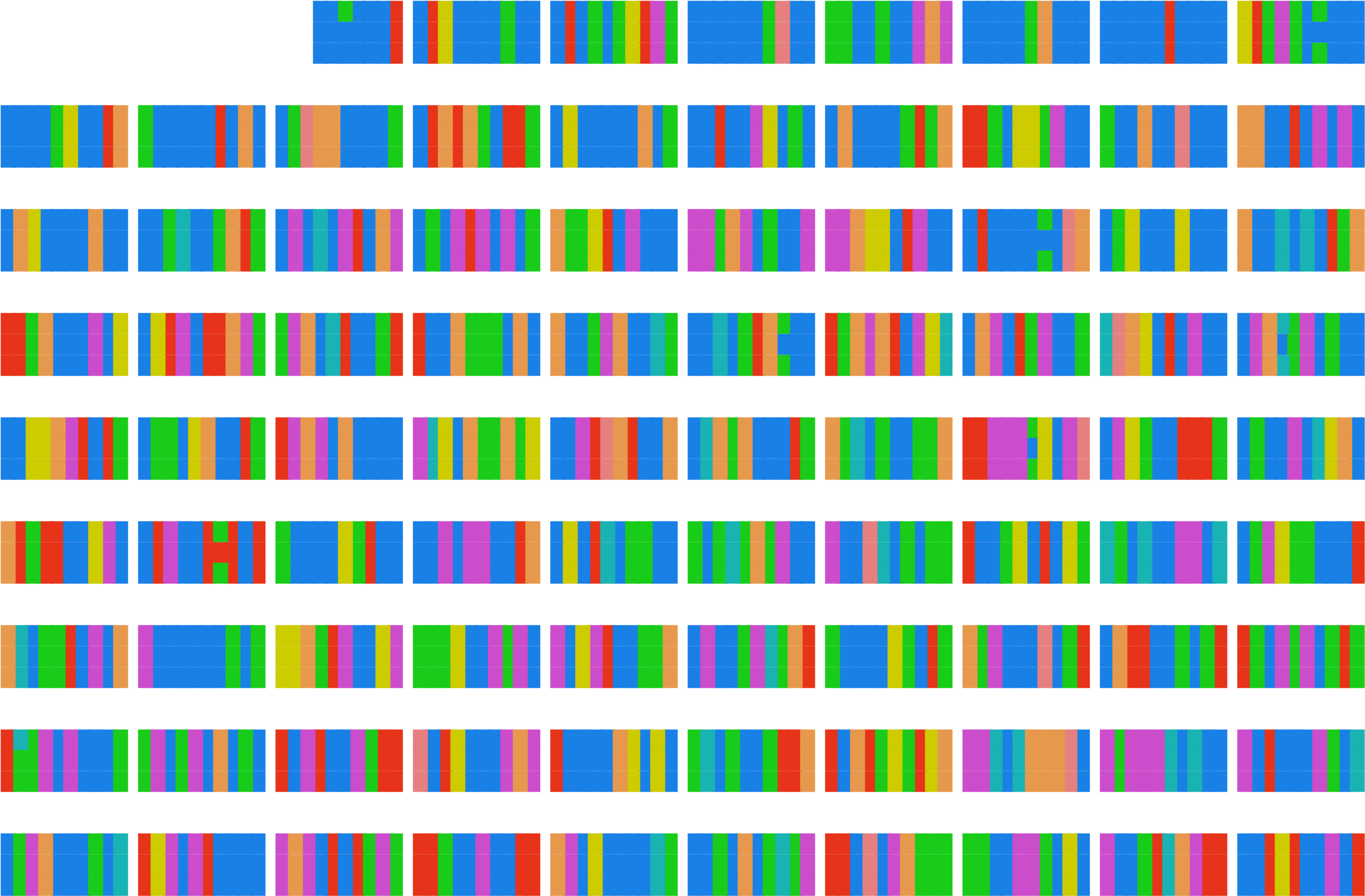
KU955592_AMR39833.1 AKLVILMGAT FAEMNTGGDV AHLALVAAFK VRPALLVSFI FRANWTPRES MLLALASCLL QTAISALEGE LMVLVNGFAL AWLAIRAMAV PRTDNIALAI KX694533_AOC50653.1 AKLAILMGAT FAEMNTGGDV AHLALIAAFK VRPALLVSFI FRANWTPRES MLLALASCLL QTVISALEGD LMVLINGFAL AWLAIRAMAV PRTDNITLAI

# 1301 KU647676_AMC33116.1 LAALTPLARG TLLVAWRAGL ATCGGFMLLS LKGKGSVKKN LPFVMALGLT AVRLVDPINV VGLLLLTRSG KRSWPPSEVL TAVGLICALA GGFAKADIEM

KU955592_AMR39833.1 LAALTPLARG TLLVAWRAGL ATCGGFMLLS LKGKGSVKKN LPFVMALGLT AVRIVDPINV VGLLLLTRSG KRSWPPSEVL TAVGLICALA GGFAKADIEM KX694533_AOC50653.1 LAALTPLARG TLLVAWRAGL ATCGGFMLLS LKGKGSVKKN LPFVMALGLT AVRLVDPINV VGLLLLTRSG KRSWPPSEVL TAVGLICALA GGFAKADIEM

# 1401 KU647676_AMC33116.1 AGPMAAVGLL IVSYVVSGKS VDMYIERAGD ITWEKDAEVT GNSPRLDVAL DESGDFSLVE DDGPPMREII LKVVLMTICG MNPIAIPFAA GAWYVYVKTG

KU955592_AMR39833.1 AGPMAAVGLL IVSYVVSGKS VDMYIERAGD ITWEKDAEVT GNSPRLDVAL DESGDFSLVE EDGPPMREII LKVVLMAICG MNPIAIPFAA GAWYVYVKTG KX694533_AOC50653.1 AGPMAAVGLL IVSYVVSGKS VDMYIERAGD ITWEKDAEVT GNSPRLDVAL DESGDFSLVE DDGPPMREII LKVVLMTICG MNPIAIPFAA GAWYVYVKTG

# 1501 KU647676_AMC33116.1 KRSGALWDVP APKEVKKGET TDGVYRVMTR RLLGSTQVGV GVMQEGVFHT MWHVTKGSAL RSGEGRLDPY WGDVKQDLVS YCGPWKLDAA WDGHSEVQLL

KU955592_AMR39833.1 KRSGALWDVP APKEVKKGET TDGVYRVMTR RLLGSTQVGV GVMQEGVFHT MWHVTKGAAL RSGEGRLDPY WGDVKQDLVS YCGPWKLDAA WDGLSEVQLL KX694533_AOC50653.1 KRSGALWDVP APKEVKKGET TDGVYRVMTR RLLGSTQVGV GVMQEGVFHT MWHVTKGSAL RSGEGRLDPY WGDVKQDLVS YCGPWKLDAA WDGHSEVQLL

# 1601 KU647676_AMC33116.1 AVPPGERARN IQTLPGIFKT KDGDIGAVAL DYPAGTSGSP ILDKCGRVIG LYGNGVVIKN GSYVSAITQG RREEETPVEC FEPSMLKKKQ LTVLDLHPGA

KU955592_AMR39833.1 AVPPGERARN IQTLPGIFKT KDGDIGAVAL DYPAGTSGSP ILDKCGRVIG LYGNGVVIKN GSYVSAITQG KREEEAPVEC FEPSMLRKKQ LTVLDLHPGA KX694533_AOC50653.1 AVPPGERARN IQTLPGIFKT KDGDIGAVAL DYPAGTSGSP ILDKCGRVIG LYGNGVVIKN GSYVSAITQG RREEETPVEC FEPSMLKKKQ LTVLDLHPGA

# 1701 KU647676_AMC33116.1 GKTRRVLPEI VREAIKTRLR TVILAPTRVV AAEMEEALRG LPVRYMTTAV NVTHSGTEIV DLMCHATFTS RLLQPIRVPN YNLYIMDEAH FTDPSSIAAR

KU955592_AMR39833.1 GKTRRVLPEI VREAIKKRLR TVILAPTRVV AAEMEEALRG LPVRYMTTAV NVTHSGTEIV DLMCHATFTS RLLQPIRVPN YNLYIMDEAH FTDPSSIAAR KX694533_AOC50653.1 GKTRRVLPEI VREAIKTRLR TVILAPTRVV AAEMEEALRG LPVRYMTTAV NVTHSGTEIV DLMCHATFTS RLLQPIRVPN YNLYIMDEAH FTDPSSIAAR

# 1801 KU647676_AMC33116.1 GYISTRVEMG EAAAIFMTAT PPGTRDAFPD SNSPIMDTEV EVPERAWSSG FDWVTDHSGK TVWFVPSVRN GNEIAACLTK AGKRVIQLSR KTFETEFQKT

KU955592_AMR39833.1 GYISTRVEMG EAAAIFMTAT PPGTRDAFPD SNSPIMDTEV EVPERAWSSG FDWVTDHSGK TIWFVPSVRN GNEIAACLTK AGKRVIQLSR KTFETEFQKT KX694533_AOC50653.1 GYISTRVEMG EAAAIFMTAT PPGTRDAFPD SNSPIMDTEV EVPERAWSSG FDWVTDHSGK TVWFVPSVRN GNEIAACLTK AGKRVIQLSR KTFETEFQKT

# 1901 KU647676_AMC33116.1 KHQEWDFVVT TDISEMGANF KADRVIDSRR CLKPVILDGE RVILAGPMPV THASAAQRRG RIGRNPNKPG DEYLYGGGCA ETDEDHAHWL EARMLLDNIY

KU955592_AMR39833.1 KNQEWDFVIT TDISEMGANF KADRVIDSRR CLKPVILDGE RVILAGPMPV THASAAQRRG RIGRNPNKPG DEYMYGGGCA ETDEDHAHWL EARMLLDNIY KX694533_AOC50653.1 KNQEWDFVVT TDISEMGANF KADRVIDSRR CLKPVILDGE RVILAGPMPV THASAAQRRG RIGRNPNKPG DEYMYGGGCA ETDEDHAHWL EARMLLDNIY

# 2001 KU647676_AMC33116.1 LQDGLIASLY RPEADKVAAI EGEFKLRTEQ RKTFVELMKR GDLPVWLAYQ VASAGITYTD RRWCFDGTTN NTIMEDSVPA EVWTRHGEKR VLKPRWMDAR KU955592_AMR39833.1 LQDGLIASLY RPEADKVAAI EGEFKLRTEQ RKTFVELMKR GDLPVWLAYQ VASAGITYTD RRWCFDGTTN NTIMEDSVPA EVWTKYGEKR VLKPRWMDAR KX694533_AOC50653.1 LQDGLIASLY RPEADKVAAI EGEFKLRTEQ RKTFVELMKR GDLPVWLAYQ VASAGITYTD RRWCFDGTTN NTIMEDSVPA EVWTRYGEKR VLKPRWMDAR 2101 KU647676_AMC33116.1 VCSDHAALKS FKEFAAGKRG AAFGVMEALG TLPGHMTERF QEAIDNLAVL MRAETGSRPY KAAAAQLPET LETIMLLGLL GTVSLGIFFV LMRNKGIGKM

KU955592_AMR39833.1 VCSDHAALKS FKEFAAGKRG AALGVMDALG TLPGHMTERF QEAIDNLAVL MRAETGSRPY KAAAAQLPET LETIMLLGLL GTVSLGIFFV LMRNKGIGKM KX694533_AOC50653.1 VCSDHAALKS FKEFAAGKRG AAFGVMEALG TLPGHMTERF QEAIDNLAVL MRAETGSRPY KAAAAQLPET LETIMLLGLL GTVSLGIFFV LMRNKGIGKM

# 2201 KU647676_AMC33116.1 GFGMVTLGAS AWLMWLSEIE PARIACVLIV VFLLLVVLIP EPEKQRSPQD NQMAIIIMVA VGLLGLITAN ELGWLERTKS DLSHLMGRRE EGATIGFSMD

KU955592_AMR39833.1 GFGMVTLGAS AWLMWLSEIE PARIACVLIV VFLLLVVLIP EPEKQRSPQD NQMAIIIMVA VGLLGLITAN ELGWLERTKS DIAHLMGRKE EGTTMGFSMD KX694533_AOC50653.1 GFGMVTLGAS AWLMWLSEIE PARIACVLIV VFLLLVVLIP EPEKQRSPQD NQMAIIIMVA VGLLGLITAN ELGWLERTKS DLGHLMGRRE EGATMGFSMD

# 2301 KU647676_AMC33116.1 IDLRPASAWA IYAALTTFIT PAVQHAVTTS YNNYSLMAMA TQAGVLFGMG KGMPFYAWDF GVPLLMIGCY SQLTPLTLIV AIILLVAHYM YLIPGLQAAA


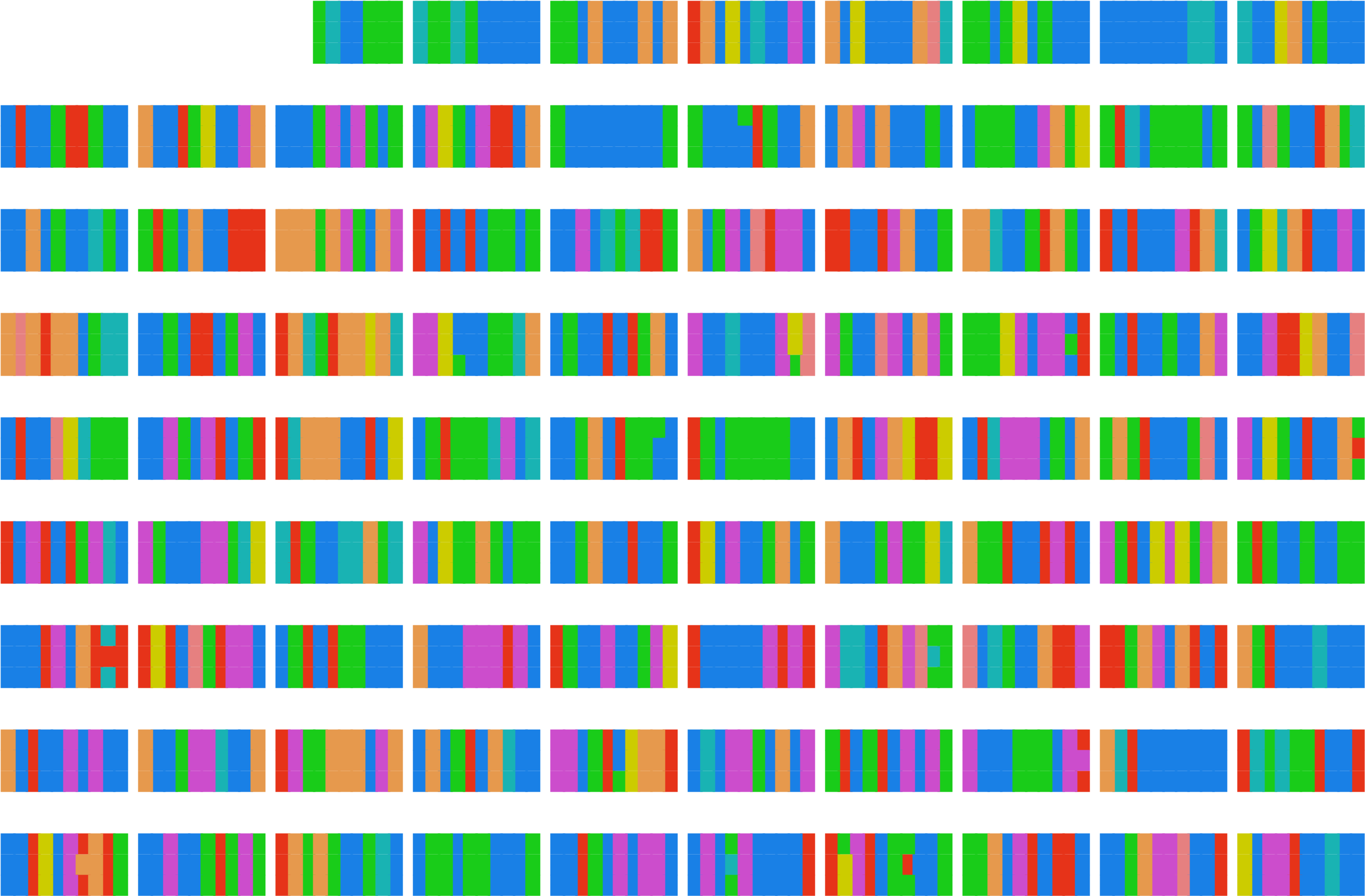
KU955592_AMR39833.1 IDLRPASAWA IYAALTTLIT PAVQHAVTTS YNNYSLMAMA TQAGVLFGMG KGMPFYAWDF GVPLLMMGCY SQLTPLTLIV AIILLVAHYM YLIPGLQAAA KX694533_AOC50653.1 IDLRPASAWA IYAALTTLIT PAVQHAVTTS YNNYSLMAMA TQAGVLFGMG KGMPFYAWDF GVPLLMMGCY SQLTPLTLIV AIILLVAHYM YLIPGLQAAA

# 2401 KU647676_AMC33116.1 ARAAQKRTAA GIMKNPVVDG IVVTDIDTMT IDPQVEKKMG QVLLIAVAVS SAILSRTAWG WGEAGALITA ATSTLWEGSP NKYWNSSTAT SLCNIFRGSY

KU955592_AMR39833.1 ARAAQKRTAA GIMKNPVVDG IVVTDIDTMT IDPQVEKKMG QVLLIAVAVS SAVLLRTAWG WGEAGALITA ATSTLWEGSP NKYWNSSTAT SLCNIFRGSY KX694533_AOC50653.1 ARAAQKRTAA GIMKNPVVDG IVVTDIDTMT IDPQVEKKMG QVLLIAVAIS SAVLLRTAWG WGEAGALITA ATSTLWEGSP NKYWNSSTAT SLCNIFRGSY

# 2501 KU647676_AMC33116.1 LAGASLIYTV TRNAGLVKRR GGGTGETLGE KWKARLNQMS ALEFYSYKKS GITEVCREEA RRALKDGVAT GGHAVSRGSA KLRWLVERGY LQPYGKVIDL

KU955592_AMR39833.1 LAGASLIYTV TRNAGLVKRR GGGTGETLGE KWKARLNQMS ALEFYSYKKS GITEVCREEA RRALKDGVAT GGHAVSRGSA KLRWLVERGY LQPHGKVVDL KX694533_AOC50653.1 LAGASLIYTV TRNAGLVKRR GGGTGETLGE KWKARLNQMS ALEFYSYKKS GITEVCREEA RRALKDGVAT GGHAVSRGSA KLRWLVERGY LQPYGKVIDL

# 2601 KU647676_AMC33116.1 GCGRGGWSYY AATIRKVQEV KGYTKGGPGH EEPVLVQSYG WNIVRLKSGV DVFHMAAEPC DTLLCDIGES SSSPEVEEAR TLRVLSMVGD WLEKRPGAFC

KU955592_AMR39833.1 GCGRGGWSYY AATIRKVQEV RGYTKGGPGH EEPMLVQSYG WNIVRLKSGV DVFHMAAEPC DTLLCDIGES SSSPEVEETR TLRVLSMVGD WLEKRPGAFC KX694533_AOC50653.1 GCGRGGWSYY AATIRKVQEV KGYTKGGPGH EEPTLVQSYG WNIVRLKSGV DVFHMAAESC DTLLCDIGES SSSPEVEEAR TLRVLSMVGD WLEKRPGAFC

# 2701 KU647676_AMC33116.1 IKVLCPYTST MMETLERLQR RYGGGLVRVP LSRNSTHEMY WVSGAKSNTI KSVSTTSQLL LGRMDGPRRP VKYEEDVNLG SGTRAVVSCA EAPNMKIIGN

KU955592_AMR39833.1 IKVLCPYTST MMETMERLQR RHGGGLVRVP LSRNSTHEMY WVSGAKSNII KSVSTTSQLL LGRMEGPRRP VKYEEDVNLG SGTRAVASCA EAPNMKIIGR KX694533_AOC50653.1 IKVLCPYTST MMETLERLQR RYGGGLVRVP LSRNSTHEMY WVSGAKSNII KSVSTTSQLL LGRMDGPRRP VKYEEDVNLG SGTRAVASCA EAPNLKIIGN

# 2801 KU647676_AMC33116.1 RIERIRSEHA ETWFFDENHP YRTWAYHGSY EAPTQGSASS LINGVVRLLS KPWDVVTGVT GIAMTDTTPY GQQRVFKEKV DTRVPDPQEG TRQVMSMVSS

KU955592_AMR39833.1 RIERIRNEHA ETWFFDENHP YRTWAYHGSY EAPTQGSASS LVNGVVRLLS KPWDVVTGVT GIAMTDTTPY GQQRVFKEKV DTRVPDPQEG TRQVMNMVSS KX694533_AOC50653.1 RVERIRSEHA ETWFFDENHP YRTWAYHGSY EAPTQGSASS LINGVVRLLS KPWDVVTGVT GIAMTDTTPY GQQRVFKEKV DTRVPDPQEG TRQVMNMVSS

# 2901 KU647676_AMC33116.1 WLWKELGKHK RPRVCTKEEF INKVRSNAAL GAIFEEEKEW KTAVEAVNDP RFWALVDKER EHHLRGECQS CVYNMMGKRE KKQGEFGKAK GSRAIWYMWL

KU955592_AMR39833.1 WLWKELGKRK RPRVCTKEEF INKVRSNAAL GAIFEEEKEW KTAVEAVNDP RFWALVDKER EHHLRGECHS CVYNMMGKRE KKQGEFGKAK GSRAIWYMWL KX694533_AOC50653.1 WLWKELGKHK RPRVCTKEEF INKVRSNAAL GAIFEEEKEW KTAVEAVNDP RFWALVDKER EHHLRGECQS CVYNMMGKRE KKQGEFGKAK GSRAIWYMWL

# 3001 KU647676_AMC33116.1 GARFLEFEAL GFLNEDHWMG RENSGGGVEG LGLQRLGYVL EEMSRIPGGR MYADDTAGWD TRISRFDLEN EALITNQMEK GHRALALAII KYTYQNKVVK

KU955592_AMR39833.1 GARFLEFEAL GFLNEDHWMG RENSGGGVEG LGLQRLGYIL EEMNRAPGGK MYADDTAGWD TRISKFDLEN EALITNQMEE GHRALALAVI KYTYQNKVVK KX694533_AOC50653.1 GARFLEFEAL GFLNEDHWMG RENSGGGVEG LGLQRLGYVL EEMSRTPGGK MYADDTAGWD TRISRFDLEN EALITNQMEK GHRALALAII KYTYQNKVVK

# 3101 KU647676_AMC33116.1 VLRPAEKGKT VMDIISRQDQ RGSGQVVTYA LNTFTNLVVQ LIRNMEAEEV LEMQDLWLLR RSEKVTNWLQ SNGWDRLKRM AVSGDDCVVK PIDDRFAHAL KU955592_AMR39833.1 VLRPAEGGKT VMDIISRQDQ RGSGQVVTYA LNTFTNLVVQ LIRNMEAEEV LEMHDLWLLR KPEKVTRWLQ SNGWDRLKRM AVSGDDCVVK PIDDRFAHAL KX694533_AOC50653.1 VLRPAERGKT VMDIISRQDQ RGSGQVVTYA LNTFTNLVVQ LIRNMEAEEV LEMQDLWLLR RPEKVTSWLQ SNGWDRLKRM AVSGDDCVVK PIDDRFAHAL 3201 KU647676_AMC33116.1 RFLNDMGKVR KDTQEWKPST GWDNWEEVPF CSHHFNKLHL KDGRSIVVPC RHQDELIGRA RVSPGAGWSI RETACLAKSY AQMWQLLYFH RRDLRLMANA

KU955592_AMR39833.1 RFLNDMGKVR KDTQEWKPST GWSNWEEVPF CSHHFNKLHL KDGRSIVVPC RHQDELIGRA RVSPGAGWSI RETACLAKSY AQMWQLLYFH RRDLRLMANA KX694533_AOC50653.1 RFLNDMGKVR KDTQEWKPST GWSNWEEVPF CSHHFNKLYL KDGRSIVVPC RHQDELIGRA RVSPGAGWSI RETACLAKSY AQMWQLLYFH RRDLRLMANA

# 3301 KU647676_AMC33116.1 ICSSVPVDWV PTGRTTWSIH GKGEWMTTED MLVVWNRVWI EENDHMEDKT PVAKWTDIPY LGKREDLWCG SLIGHRPRTT WAENIKNTVN MVRRIIGDEE

KU955592_AMR39833.1 ICSAVPIDWV PTGRTTWSIH GKGEWMTTED MLMVWNRVWI EENDHMEDKT PVTKWTDIPY LGKREDLWCG SLIGHRPRTT WAENIKDTVN MVRRIIGDEE KX694533_AOC50653.1 ICSSVPVDWV PTGRTTWSIH GKGEWMTTED MLVVWNRVWI EENDHMEDKT PVTKWTDIPY LGKREDLWCG SLIGHRPRTT WAENIKDTVN MVRRIIGDEE

# 3401 KU647676_AMC33116.1 KYMDYLSTQV RYLGEEGSTP GVL KU955592_AMR39833.1 KFMDYLSTQV RYLGEEGSTP GVL KX694533_AOC50653.1 KYMDYLSTQV RYLGEEGSTP GVL
