# Supplementary material for: Experimental infections with Zika virus strains reveal high vector competence of Aedes albopictus and Aedes aegypti populations from Gabon (Central Africa) for the African virus lineage
Source: Emerg Microbes Infect. 2021 Jun 18;10(1):1244–53. doi: 10.1080/22221751.2021.1939167 (PMC8216262; doi:10.1080/22221751.2021.1939167)
Supplement: Supplementary_information_Jiolle_et_al._r_vis_e_clean_file.docx [file TEMI_A_1939167_SM6361.docx]

**Supplementary information**

**Experimental infections with Zika virus strains reveal high vector competence of *Aedes albopictus* and *Aedes aegypti* populations from Gabon (Central Africa) for the African virus lineage**

Davy Jiolle^1^, Isabelle Moltini-Conclois^1^, Judicaël Obame-Nkoghe^2,3^, Patrick Yangari^2^, Angélique Porciani^1^, Bethsabée Scheid^1^, Kengne Pierre^1,2^, Diego Ayala1^1,2^, Anna-Bella Failloux^4^, Christophe Paupy^1*^

^1^MIVEGEC Laboratory, Montpellier Univ., IRD, CNRS., Montpellier, 34394, France

^2^Centre Interdisciplinaire de Recherches Médicales de Franceville, Franceville, Gabon

^3^Laboratoire de Biologie Moléculaire et Cellulaire, Département de Biologie, Université des Sciences et Techniques de Masuku, Franceville, Gabon

^4^Institut Pasteur, Department of Virology, Unit of Arboviruses and Insect Vectors, Paris, France

**Correspondence**: Christophe Paupy

**E-mail**: [christophe.paupy@ird.fr](mailto:christophe.paupy@ird.fr)

**Running title**: *Aedes aegypti* and *Aedes albopictus* from Gabon better transmit African Zika virus

1. **Procedure for mosquito sampling and rearing**

Wild eggs (i.e. F0 generation) of *Aedes* mosquitoes were collected using ovitraps deployed in five sites of Franceville (Ayassi, CIRMF, Menaye, Mangoungou, and Mbaya, March 2018) and in three sites of Libreville (Akanda, Sainte-Marie and Sibang, August 2018). Ovitraps (ten per site) consisted of 1L black plastic cups lined with a 38 × 15 cm piece of seed germination paper (Anchor Paper Co.) and filled with 3–8 cm of water. Germination papers were renewed every 3 days for 12 days. After drying, egg-impregnated papers were shipped to IRD, Montpellier, France, for egg hatching and larval breading in osmotic water to obtain adults that were sorted by species and housed in 30×30×30 cm screened cages with permanent access to a 10% sucrose solution. Three composite populations were established, *Ae. albopictus* from Franceville (FCV), *Ae. aegypti* from Franceville and *Ae. albopictus* from Libreville (LBV), by mixing male and female adults. Mosquitoes were maintained in controlled insectary conditions (28°C, 80% relative humidity, 14:10 hour light-dark cycle) by mass rearing and collective oviposition. Adult females were fed with rabbit blood through a membrane feeding system (Hemotek Ltd, Blackburn, UK) using porcine intestine as membrane.

1. **Protocol for the ZIKV production**

Sub-confluent Vero cells (CCL-81) in a 75-cm^2^ culture flask were infected with 500µl of 0.1 moi inoculum and incubated at 37°C and 5% CO_2_ for 1h. Then, 10ml of DMEM supplemented with 0.1% penicillin (10,000 U/ml)/streptomycin (10,000 µg/ml), 2% of decomplemented foetal bovine serum (FBS) was added. After 3-5 days of incubation (Table S1), the cell culture medium was harvested, centrifuged at 3,000g for 5min, and adjusted to 10% FBS and to pH ~8 (with 1% of 7.5% sodium bicarbonate). Viruses were concentrated about 10 times using Amicon Ultra-15 Centrifugal Filter Devices 100K (Millipore) to reach at least 3 x 10^6^ PFU/mL. Virus stocks were aliquoted and stored at -80°C. Virus titration was performed by standard plaque assay.

1. **Plaque assay protocol used for ZIKV detection in mosquito body and heads, and quantification in saliva.**

Vero cells were seeded in a 96-well plate and grown overnight. Bodies and heads were directly grind in their 2mL tubes at 10m/sec (FastPrep-24 5G, MP Biomedicals) for 30s, and centrifuged at 10.000g at 4°C for 5min. The culture medium was removed and cells were inoculated with 100μL of serial diluted sample. After incubation at 37°C and 5% CO_2_ for 1h, the inoculum was removed and cells were covered with 150 μL/well of a 1:1 mix of overlay medium (DMEM, 0.1% 2x penicillin/streptomycin, 2x antibiotic-antimycotic (Life Technologies) and 4% FBS) and 3.2% methylcellulose (Sigma-Aldrich, St Louis, MO, USA) solution, and incubated at 37°C and 5% CO_2_ for 5 days. Cells were fixed at room temperature (20-25°C) by adding 150μL/well of 3.6% formaldehyde in phosphate-buffered saline (PBS) for 30 min. Then, cells were washed three times with PBS, and incubated with 150μL/well of 0.4% crystal violet (Sigma-Aldrich) in 3% formaldehyde at room temperature for 45min. After three washes in water, ZIKV presence was observed. ZIKV presence in saliva was determined by inoculating 25µL of saliva in 6-well plates, and by counting foci by the naked eye and converting them into PFU/saliva.

**Table S1:** Main characteristics of the Zika virus strains used for experimental mosquito infections.

|  | **Virus name (abbreviation)** |  | **Lineage** |  | **Strain/Geographical Origin/ Isolation date** |  | **Isolation host** |  | **Provider** |  | **Passage history since isolation** |  | **Production cell Line** |  | **Passage** |  | **Incubation period (in days)** |  | **Stock virus titre (concentrated?)** |  | **Titration method** |  |
| --- | --- | --- | --- | --- | --- | --- | --- | --- | --- | --- | --- | --- | --- | --- | --- | --- | --- | --- | --- | --- | --- | --- |
|  | Zika Dakar (DAK84) |  | African |  | DAK84/Senegal/1984 |  | *Aedes taylori* |  | EVAg |  | AP61 (1), C6/36 (1), Vero (3), BHK-21 (4) |  | Vero CCL-81 |  | 3 |  | 3 |  | 8.40 x 10^7^PFU/ml (Yes) |  | Plaque assay |  |
|  | Zika Martinique (MARTI) |  | Asian |  | OPY_Martinique_PaRi_2015/Martinique/2015 |  | Human serum |  | EVAg |  | Vero (3) |  | Vero CCL-81 |  | 3 |  | 3 |  | 3.34 x 10^7^PFU/ml (Yes) |  | Plaque assay |  |
|  | Zika Malaysia (MAS66) |  | Asian |  | MAS66/Malaysia/1966 |  | *Aedes aegypti* |  | EVAg |  | SM (6), BHK-21 (1), C6/36 (1), Vero (2), Vero E6 (5) |  | Vero CCL-81 |  | 5 |  | 5 |  | 1.16 x 10^8^PFU/ml (Yes) |  | Plaque assay |  |

**Table S2:** NS1 variability in the three ZIKV strains used for experimental infections of mosquitoes.

|  |  | **Variable positions*** | | | | |
| --- | --- | --- | --- | --- | --- | --- |
| **ZIKV strain** |  | **982** | **988** | **1007** | **1030** | **1118** |
| **MARTI** |  | V | V | K | I | **W** |
| **DAK84** |  | V | **A** | **R** | **V** | R |
| **MAS66** |  | **A** | V | K | I | R |

*: Position in the polyprotein (NS1 from 797 to 1148)

**Table S3:** Significant pairwise comparisons of VC parameters (IR, DE and TE) assessed at 7, 14 and 21 dpi for three ZIKV strains in three mosquito populations from Gabon.

|  | **VC parameter** |  | **Time point** |  | **Mosquito/ Virus strain** |  | **Virus/ Mosquito pair** |  | **Test** |  | **p-value** |  |
| --- | --- | --- | --- | --- | --- | --- | --- | --- | --- | --- | --- | --- |
|  |  |  |  |  |  |  |  |  |  |  |  |  |
|  | **Infection Rate** |  | 7 dpi |  | *Ae. albopictus* FCV |  | DAK84-MARTI |  | Tuckey |  | 0.00212 |  |
|  |  |  |  |  | *Ae. albopictus* FCV |  | DAK84-MAS66 |  | Tuckey |  | 0.00212 |  |
|  |  |  |  |  | *Ae. albopictus* LBV |  | DAK84-MARTI |  | Tuckey |  | 0.00002 |  |
|  |  |  |  |  | *Ae. albopictus* LBV |  | DAK84-MAS66 |  | Tuckey |  | 0.00009 |  |
|  |  |  |  |  | *Ae. aegypti* FCV |  | DAK84-MARTI |  | Tuckey |  | 0.00097 |  |
|  |  |  |  |  | *Ae. aegypti* FCV |  | DAK84-MAS66 |  | Tuckey |  | 0.00001 |  |
|  |  |  |  |  |  |  |  |  |  |  |  |  |
|  |  |  | 14 dpi |  | *Ae. albopictus* FCV |  | DAK84-MARTI |  | Tuckey |  | 0.00022 |  |
|  |  |  |  |  | *Ae. albopictus* FCV |  | DAK84-MAS66 |  | Tuckey |  | 0.00002 |  |
|  |  |  |  |  | *Ae. albopictus* LBV |  | DAK84-MARTI |  | Tuckey |  | 0.00002 |  |
|  |  |  |  |  | *Ae. albopictus* LBV |  | DAK84-MAS66 |  | Tuckey |  | 0.00004 |  |
|  |  |  |  |  | *Ae. aegypti* FCV |  | DAK84-MARTI |  | Tuckey |  | 0.00001 |  |
|  |  |  |  |  | *Ae. aegypti* FCV |  | DAK84-MAS66 |  | Tuckey |  | 0.00001 |  |
|  |  |  |  |  |  |  |  |  |  |  |  |  |
|  |  |  | 21 dpi |  | *Ae. albopictus* FCV |  | DAK84-MARTI |  | Tuckey |  | 0.03618 |  |
|  |  |  |  |  | *Ae. albopictus* FCV |  | DAK84-MAS66 |  | Tuckey |  | 0.00200 |  |
|  |  |  |  |  | *Ae. albopictus* LBV |  | DAK84-MARTI |  | Tuckey |  | 0.00001 |  |
|  |  |  |  |  | *Ae. albopictus* LBV |  | DAK84-MAS66 |  | Tuckey |  | 0.00001 |  |
|  |  |  |  |  | *Ae. aegypti* FCV |  | DAK84-MARTI |  | Tuckey |  | 0.00045 |  |
|  |  |  |  |  | *Ae. aegypti* FCV |  | DAK84-MAS66 |  | Tuckey |  | 0.00001 |  |
|  |  |  |  |  | DAK84 |  | *Ae. albopictus* FCV-*Ae. albopictus-*LBV |  | Tuckey |  | 0.02119 |  |
|  |  |  |  |  |  |  |  |  |  |  |  |  |
|  | **Dissemination Efficiency** |  | 7 dpi |  | *Ae. albopictus* LBV |  | DAK84-MARTI |  | Tuckey |  | 0.01267 |  |
|  |  |  |  |  | *Ae. albopictus* LBV |  | DAK84-MAS66 |  | Tuckey |  | 0.01267 |  |
|  |  |  |  |  | *Ae. aegypti* FCV |  | DAK84-MARTI |  | Tuckey |  | 0.02705 |  |
|  |  |  |  |  | *Ae. aegypti* FCV |  | DAK84-MAS66 |  | Tuckey |  | 0.01246 |  |
|  |  |  |  |  | DAK84 |  | *Ae. albopictus* FCV-*Ae. aegypti-*FCV |  | Tuckey |  | 0.04551 |  |
|  |  |  |  |  | DAK84 |  | *Ae. albopictus* FCV-*Ae. albopictus-*LBV |  | Tuckey |  | 0.00765 |  |
|  |  |  |  |  |  |  |  |  |  |  |  |  |
|  |  |  | 14 dpi |  | *Ae. albopictus* FCV |  | DAK84-MARTI |  | Tuckey |  | 0.00212 |  |
|  |  |  |  |  | *Ae. albopictus* FCV |  | DAK84-MAS66 |  | Tuckey |  | 0.00008 |  |
|  |  |  |  |  | *Ae. albopictus* LBV |  | DAK84-MARTI |  | Tuckey |  | 0.00001 |  |
|  |  |  |  |  | *Ae. albopictus* LBV |  | DAK84-MAS66 |  | Tuckey |  | 0.00001 |  |
|  |  |  |  |  | *Ae. aegypti* FCV |  | DAK84-MARTI |  | Tuckey |  | 0.00002 |  |
|  |  |  |  |  | *Ae. aegypti* FCV |  | DAK84-MAS66 |  | Tuckey |  | 0.00002 |  |
|  |  |  |  |  |  |  |  |  |  |  |  |  |
|  |  |  | 21 dpi |  | *Ae. albopictus* FCV |  | DAK84-MAS66 |  | Tuckey |  | 0.02110 |  |
|  |  |  |  |  | *Ae. albopictus* LBV |  | DAK84-MARTI |  | Tuckey |  | 0.00001 |  |
|  |  |  |  |  | *Ae. albopictus* LBV |  | DAK84-MAS66 |  | Tuckey |  | 0.00001 |  |
|  |  |  |  |  | *Ae. aegypti* FCV |  | DAK84-MARTI |  | Tuckey |  | 0.00045 |  |
|  |  |  |  |  | *Ae. aegypti* FCV |  | DAK84-MAS66 |  | Tuckey |  | 0.00001 |  |
|  |  |  |  |  | DAK84 |  | *Ae. albopictus* FCV-*Ae. aegypti-*FCV |  | Tuckey |  | 0.01579 |  |
|  |  |  |  |  | DAK84 |  | *Ae. albopictus* FCV-*Ae. albopictus-*LBV |  | Tuckey |  | 0.00229 |  |
|  |  |  |  |  |  |  |  |  |  |  |  |  |
|  | **Transmission Efficiency** |  | 14 dpi |  | *Ae. albopictus* FCV |  | DAK84-MARTI |  | Tuckey |  | 0.00739 |  |
|  |  |  |  |  | *Ae. albopictus* FCV |  | DAK84-MAS66 |  | Tuckey |  | 0.00739 |  |
|  |  |  |  |  | *Ae. albopictus* LBV |  | DAK84-MARTI |  | Tuckey |  | 0.00010 |  |
|  |  |  |  |  | *Ae. albopictus* LBV |  | DAK84-MAS66 |  | Tuckey |  | 0.00008 |  |
|  |  |  |  |  | *Ae. aegypti* FCV |  | DAK84-MARTI |  | Tuckey |  | 0.00004 |  |
|  |  |  |  |  | *Ae. aegypti* FCV |  | DAK84-MAS66 |  | Tuckey |  | 0.00147 |  |
|  |  |  |  |  |  |  |  |  |  |  |  |  |
|  |  |  | 21 dpi |  | *Ae. albopictus* FCV |  | DAK84-MAS66 |  | Tuckey |  | 0.01246 |  |
|  |  |  |  |  | *Ae. albopictus* FCV |  | DAK84-MAS66 |  | Tuckey |  | 0.02705 |  |
|  |  |  |  |  | *Ae. albopictus* LBV |  | DAK84-MARTI |  | Tuckey |  | 0.00001 |  |
|  |  |  |  |  | *Ae. albopictus* LBV |  | DAK84-MAS66 |  | Tuckey |  | 0.00001 |  |
|  |  |  |  |  | *Ae. aegypti* FCV |  | DAK84-MARTI |  | Tuckey |  | 0.00004 |  |
|  |  |  |  |  | *Ae. aegypti* FCV |  | DAK84-MAS66 |  | Tuckey |  | 0.00004 |  |
|  |  |  |  |  | DAK84 |  | *Ae. albopictus* FCV-*Ae. aegypti-*FCV |  | Tuckey |  | 0.03432 |  |
|  |  |  |  |  | DAK84 |  | *Ae. albopictus* FCV-*Ae. albopictus-*LBV |  | Tuckey |  | 0.00830 |  |
|  |  |  |  |  |  |  |  |  |  |  |  |  |

**Legend for Figure S1:** Saliva titres for the three mosquito populations according to the ZIKV strain (MARTI, DAK84, or MAS66) and incubation time (all time points combined, 7, 14 and 21 days post-infection). Each coloured dot represents the titre of a saliva sample. Black dots and vertical bars represent the mean (± SE). p: probability associated with the global test (Kruskal-Wallis) to compare the mean viral loads in the different mosquito populations (in bold when <0.05). Horizontal bars and the associated annotation correspond to pairwise comparisons of the mean viral loads (Dunn test) done when the global test was significant. NA: not applicable; NS: not significant (p>0.05); *: p < 0.05; **: p < 10^-2^; ***: p< 10^-3^.

**Legend for Figure S2:** Alignment of the polyprotein sequences of the MARTI (KU647676), DAK84 (KU955592) and MAS66 (KX694533) ZIKV strains.
